# FANCM Gene Variants in a Male Diagnosed with Sertoli Cell-Only Syndrome and Diffuse Astrocytoma

**DOI:** 10.3390/genes15060707

**Published:** 2024-05-28

**Authors:** Monika Logara Klarić, Tihana Marić, Lucija Žunić, Lovro Trgovec-Greif, Filip Rokić, Ana Fiolić, Ana Merkler Šorgić, Davor Ježek, Oliver Vugrek, Antonia Jakovčević, Maja Barbalić, Robert Belužić, Ana Katušić Bojanac

**Affiliations:** 1 Genom Ltd., Ilica 190, 10000 Zagreb, Croatia; monika@genom.hr (M.L.K.); lucija@genom.hr (L.Ž.); ana@genom.hr (A.F.); maja@genom.hr (M.B.); 2 Department of Medical Biology, School of Medicine, University of Zagreb, Šalata 3, 10000 Zagreb, Croatia; tihana.maric0@gmail.com; 3 Center of Excellence for Reproductive and Regenerative medicine, School of Medicine, University of Zagreb, 10000 Zagreb, Croatia; amerkler@kbc-zagreb.hr (A.M.Š.); davor.jezek@mef.hr (D.J.); 4 Laboratory for Advanced Genomics, Division of Molecular Medicine, Rudjer Boskovic Institute, Bijenička Cesta 54, 10000 Zagreb, Croatia; lovro.lovro@hotmail.com (L.T.-G.); frokic@irb.hr (F.R.); ovugrek@irb.hr (O.V.); 5 Department of Histology and Embryology, School of Medicine, University of Zagreb, Šalata 3, 10000 Zagreb, Croatia; 6 Department of Pathology, University Hospital Center Zagreb, Kišpatićeva 12, 10000 Zagreb, Croatia; 7 Faculty of Science, University of Split, Rudjera Bošković 33, 21000 Split, Croatia; 8 Laboratory for Metabolism and Aging, Division of Molecular Medicine, Rudjer Boskovic Institute, Bijenička Cesta 54, 10000 Zagreb, Croatia; robert.beluzic@irb.hr

**Keywords:** non-obstructive azoospermia, male infertility, SCOS syndrome, FANCM, astrocytoma, cancer

## Abstract

Azoospermia is a form of male infertility characterized by a complete lack of spermatozoa in the ejaculate. Sertoli cell-only syndrome (SCOS) is the most severe form of azoospermia, where no germ cells are found in the tubules. Recently, FANCM gene variants were reported as novel genetic causes of spermatogenic failure. At the same time, FANCM variants are known to be associated with cancer predisposition. We performed whole-exome sequencing on a male patient diagnosed with SCOS and a healthy father. Two compound heterozygous missense mutations in the FANCM gene were found in the patient, both being inherited from his parents. After the infertility assessment, the patient was diagnosed with diffuse astrocytoma. Immunohistochemical analyses in the testicular and tumor tissues of the patient and adequate controls showed, for the first time, not only the existence of a cytoplasmic and not nuclear pattern of FANCM in astrocytoma but also in non-mitotic neurons. In the testicular tissue of the SCOS patient, cytoplasmic anti-FANCM staining intensity appeared lower than in the control. Our case report raises a novel possibility that the infertile carriers of FANCM gene missense variants could also be prone to cancer development.

## 1. Introduction

Male infertility affects 7–12% of men in the general population [1,2]. Azoospermia is the most severe male infertility condition, with the complete absence of spermatozoa in the ejaculate (WHO 2010), and it is present in 12–22% of infertile males [3,4]. One of the most severe testicular pathologies is Sertoli cell-only syndrome (SCOS), described as a complete absence of germ cells in tubules that contain only Sertoli cells [5,6]. Up to 80% of patients with azoospermia can be classified by the underlying cause of the condition, which includes physical obstruction of the genital tract; genetic abnormalities such as chromosome Y microdeletions; Klinefelter syndrome; CFTR mutations; and other hormonal and testicular anomalies [7,8]. It is widely speculated that rare genetic events might cause the remaining unexplained non-obstructive azoospermia (NOA) in men [9].

Astrocytomas are primary human brain tumors originating in astrocytes, star-shaped glial cells in the cerebrum, which include diffuse or anaplastic astrocytomas that develop into secondary glioblastomas over several months or years. [10,11]. Astrocytomas typically occur sporadically, but a genetic predisposition for gliomas is likely to exist [12]. Also, their genetic association with some cancer susceptibility syndromes linked to aberrant DNA repairs have been shown, such as neurofibromatosis type I, Li-Fraumeni syndrome, Turcot syndrome, and tuberous sclerosis [13].

Considering somatic mutational burden, the vast majority of histologically diagnosed astrocytomas exhibit a characteristic mutation of the isocitrate dehydrogenase 1/2 gene (IDH1/2), which points to a risk of progression. [14]. Another gene important for astrocytoma stratification is ATRX (Alpha Thalassemia/Mental Retardation Syndrome X-Linked), whose expressional loss often co-occurs with IDH mutations and in younger patients [15,16]. ATRX is an epigenetic regulator important for DNA damage responses (DDR) but is also involved in the suppression of alternative telomere lengthening during cell replication (Alternative Lengthening of Telomeres, ALT) [17] seen in 10–15% of glioma cases [18]. Interestingly, ALT is also inhibited by DNA translocase from Fanconi Anemia (FA) Complementation Group M (FANCM). It is a member of the Fanconi anemia (FA) gene family. [19,20], which restricts telomeric replication stress by interacting with the BTR (BLM-TOP3A-RMI) complex. [21,22]. FANCM has been shown to contribute to genomic stability. The primary role of FANCM is to assist in DNA repair, DNA replication, and DNA interstrand crosslink repair. It acts as a tumor suppressor gene by suppressing spontaneous sister chromatid exchanges and maintaining chromosomal stability [23,24]. Altogether, any decrease in ATRX or FANCM function could impair genome stability and contribute to telomere maintenance by ALT and, consequently, cancer development or progression.

In humans, the putative involvement of FANCM gene variants in male and female fertility has been described in several families. Male patients carrying biallelic mutations in FANCM exhibit sterility characterized by non-obstructive azoospermia and Sertoli cell-only syndrome [25,26,27,28]. Considering cancer, FANCM has also been reported to be a tumor suppressor and cancer-susceptibility gene [29,30], with its germline variants related to breast cancer [31,32,33,34] but also to some other cancers, such as B-cell precursor lymphoblastic leukemia or squamous cell carcinoma [28,35,36,37].

In this study, we present a 38-year-old patient seeking infertility treatment who was screened by whole-exome sequencing for azoospermia-related genes as a part of a research study. The patient subsequently developed cancer, a diffuse astrocytoma. We sought to determine the contribution of this infertility-related gene to its clinical pattern in azoospermic testes but also in brain cancer.

## 2. Materials and Methods

### 2.1. Sample

Due to idiopathic infertility, a 38-year-old azoospermic patient was referred to the University Hospital Zagreb for a testicular sperm extraction (TESE) procedure, which includes an open surgical biopsy of both testes and testicular histology examination. The latter showed classical histology of Sertoli cell-only syndrome with an absence of spermatogonia, spermatocytes, or spermatids in both testes, with some tubules exhibiting fibrotic changes (tubular fibrosis/tubular ‘shadows’) in the left testis. Known causal factors for male infertility, like Y AZF microdeletions or Klinefelter syndrome, were not detected. Subsequent to the above infertility processing, the patient was diagnosed with a central nervous system tumor, classified as a diffuse astrocytoma. There was no history of genetic diseases or infertility in his family. Both the patient and his parents voluntarily participated in this research. According to the study protocol, the patient and his parents provided written informed consent.

### 2.2. Whole-Exome Sequencing and Bioinformatic Analysis

Whole-exome sequencing was performed on the index patient and his healthy father.

Genomic DNA was extracted from the participants’ peripheral blood samples using a standard extraction procedure (Invitrogen™ iPrep™ PureLink™ gDNA Blood Kit Thermo Fisher Scientific Inc. Waltham, Massachusetts, United States). Whole-exome sequencing was performed by Macrogen Inc. using the NovaSeq6000 platform and the Agilent SureSelect XT_V5+UTR library preparation kit.

Obtained sequences were aligned to human reference genome version hg38 [38] using the BWA-MEM software [39]. Variants were called with the HaplotypeCaller algorithm from GATK [40] and annotated with Jannovar [41] using the transcript definition database from UCSC [42].

All variants with population frequency higher than 1% in ExAC database 0.3 for GRCh38 [43] and GnomAD database release 2.1.1 [44], as well as intronic and synonymous variants, were removed from both samples’ data. Filtered data were then assessed using the Exomiser software, which prioritizes genes and variants relative to phenotype matches with the known phenotype of diseased genes from human and model organism data [45]. The human phenotype ontology terms used in Exomiser were male infertility (HP:000325) and non-obstructive azoospermia (HP:0011961). The Exomizer pedigree option was used for analysis in order to track the segregation pattern from the fertile parents to the infertile son.

**Figure 1:**
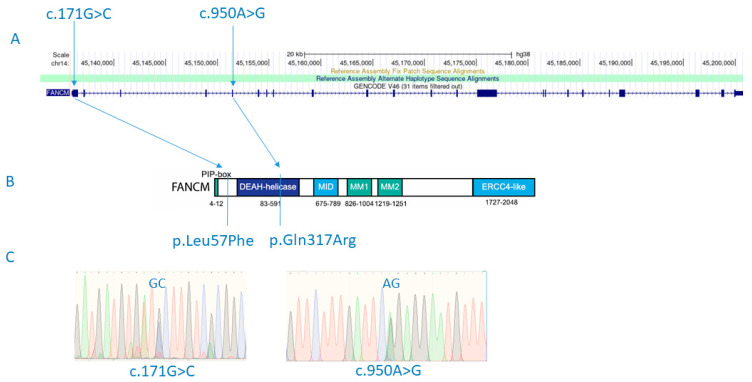
FANCM mutations (c.171G>C and c.950A>G) detected in a male patient with SCOS and diffuse astrocytoma are shown in the context of relative positions in the gene (**A**) and the protein structure (p.Leu57Phe and p.Gln317Arg) (**B**). Detected variants shown in electropherogram diagrams after experimental validation using Sanger sequencing (**C**). Figure 1B adapted from Poole, L. A., & Cortez, D. (2017). Functions of SMARCAL1, ZRANB3, and HLTF in maintaining genome stability. *Critical reviews in biochemistry and molecular biology*, *52*(6), 696–714. https://doi.org/10.1080/10409238.2017.1380597.

Sanger sequencing of candidate variants found in the FANCM gene was performed on the patient and his parents for variant validation, as well as to assess the segregation pattern of FANCM variants detected in the patient (Figure 1C).

### 2.3. Immunohistochemical Detection of FANCM Protein

Immunohistochemical (IHC) detection of FANCM protein was performed on the patient’s formalin-fixed paraffin-embedded (FFPE) biopsies of testicular tissue and astrocytoma, before and after radiotherapy. Testicular samples with complete spermatogenesis and prostate tissue or astrocytoma biopsies from two patients with similar characteristics to the SCOS patient were used as controls (IDH1 R132H+, ATRX-positive, p53-negative, and low proliferative index). All FFPE biopsies were obtained from the Department of Pathology and Cytology of the University Hospital Zagreb.

Anti-human FANCM antibodies (1:100, CV5.1; Novus Biologicals LLC a Bio-Techne Brand, Zillow, USA 1507–1679; 1:150 rabbit anti-human antibodies, aa 268–288 and aa 2002–2019 (a kind gift from Fanconi Anemia Research Fund)) were used for the detection of FANCM on testicular and astrocytoma microscopic slides after overnight incubation, while DNA breaking points were visualized with the anti-γH2AX antibody (1:500, ab81299; Abcam, Biomedical Campus, Discovery Dr, Trumpington, Cambridge CB2 0AX, UK) After the application of appropriate HRP-conjugated donkey anti-mouse (ab6820; Abcam, UK) or goat anti-rabbit (ab97051; Abcam, Biomedical Campus, Discovery Dr, Trumpington, Cambridge CB2 0AX, UK) secondary antibodies for 60 min on RT, signal visualization was performed using Liquid DAB and a Substrate Chromogen System (Dako, Agilent Technologies, 5301 Stevens Creek Blvd Santa Clara, CA, USA) according to the manufacturer’s instructions.

All immunohistochemical analyses were performed by an experienced andrologist and clinical pathologist using a Nikon DS-Fi2 microscope and the Nikon NIS Elements F. 400.06 software.

## 3. Results

### 3.1. Characterization of the Patient

The patient was diagnosed with non-obstructive azoospermia, specifically, with Sertoli cell-only syndrome (SCOS), with tubules containing only Sertoli cells or showing tubular fibrosis (Supplementary Figure S1). In testicular biopsies, inflammatory infiltrates of mononuclear cells were detected, while Leydig cells appeared normal without signs of a hyperplastic/hypertrophic cellular phenotype and did not form micronodules. The patient had FSH and LH levels in the normal range (11.5 versus reference 1.5–12.4 U/I; 5.5 versus reference 1.7–8.6, respectively). Testosterone levels were below the reference value (9.7 versus reference 9.9–27.8). The patient had no history of testis varicocele or cryptorchidism; deletions in the AZF region; or mutations in the CFTR gene, and his karyotype showed normal male (46, XY).

Subsequent to infertility, the patient developed a diffuse astrocytoma, WHO grade II, with two focal lesions. Atypical glial cells with astroglial morphology and hyperchromatic nuclei were found (Supplementary Figure S2). Molecular immunohistochemical analysis of the astrocytoma showed a common mutation in the IDH1 gene, a substitution of arginine into histidine (R132H) in the exon, and a common negative mutation in the ATRX gene, which is associated with good prognosis and treatment sensitivity. The astrocytoma was p53-negative, exhibiting low proliferative capacity (Ki67 = 2%) and no detectable mitosis or necrosis. The blood vessels had no sign of endothelial proliferation. The patient underwent radiotherapy; however, 9 months later, a control MRI indicated a tumor transformation. After another surgery, a subsequent histopathological analysis of the second biopsy revealed a hypocellular diffuse astrocytoma with the same features as previously described but decreased in size. In addition, primarily reactive tumor cells were present, with areas of post-radiation histological changes such as chronic perivascular lymphocytes, histocyte infiltration, and gliosis.

### 3.2. Identification of Potentially Pathogenic Variants from WES

The patient and his healthy father underwent whole-exome sequencing (WES) for an infertility research project. Exome sequencing yielded 1.156.950 high-quality variants in the son and 1.083.777 in the father. The data for each sample were filtered to remove all variants with minor allele frequency greater than 1% and all noncoding and synonymous variants that resulted in 2061 variants in the son and 2591 variants in the father. The remaining variants were analyzed by the Exomiser software, taking into account the segregation pattern of the fertile father and the infertile son and using human phenotype ontology (HPO) terms for male infertility and non-obstructive azoospermia for phenotype matches.

Variants with an Exomiser combined score greater than 0.9 are shown in Supplementary Table S1. The highest Exomiser score (0.939) was obtained for the FANCM gene for a recessive inheritance model. In particular, it was shown that the son had a compound heterozygote for two missense variants (p.Gln317Arg; rs375644492/p.Leu57Phe; rs142007602) in the FANCM gene (Figure 1). Both variants were confirmed by Sanger sequencing. Variant rs375644492 was shared with the healthy father, while the variant rs142007602 was inherited from the mother. The minor allele frequency (MAF) is 0.00001593 for rs375644492 and 0.001738 for rs142007602 in GnomAD v2.1.1. The Combined Annotation-Dependent Depletion (CADD) for rs375644492 is 19.4, and for rs142007602, it is 21.4. The current classification by ClinVar for rs375644492 is of uncertain significance, while variant rs142007602 shows conflicting interpretations of pathogenicity: it is classified as probably benign regarding Fanconi anemia, but in relation to spermatogenic failure, it is considered of uncertain significance. Additionally, Sift and MutationTaster both classify rs142007602 as ‘deleterious’.

### 3.3. Localization of FANCM in Testicular Tissue

**Figure 2:**
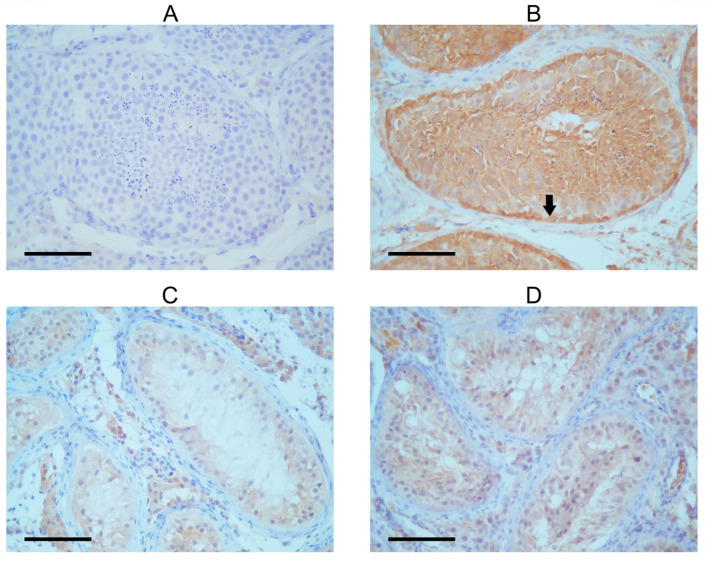
Immunohistochemical detection of FANCM in tubules of SCOS patient and control with complete spermatogenesis. (**A**) Negative control. (**B**) Seminiferous tubule with complete spermatogenesis; the black arrow shows strong FANCM expression in the cytoplasm of spermatogenic cells, especially in the spermatogonia near the tubular wall. Signal intensity increases as spermatogenesis progresses. Sertoli cells show a weak FANCM staining signal. The cytoplasm of Leydig cells is also positive. (**C**,**D**) Tubules of SCOS patients show very faintly stained Sertoli cell cytoplasm, but only tubules and interstitial Leydig cells. Spermatogenic cells are lacking.

The seminiferous tubules of the patient exhibited a very low presence of FANCM in the immunohistochemical detection, with Sertoli cells being only faintly stained (Figure 2). This finding is in concordance with previously published work by Kasak et al. [14]. A control testis with complete spermatogenesis showed intensive cytoplasmic FANCM staining in the tubular compartment (Table 1). The strongest staining intensity was observed in the cytoplasm of spermatogonia lining the tubular walls. Moderate cytoplasmic staining was shown in primary spermatocytes and spermatids in the apical compartment of the seminiferous epithelium. The expression of FANCM was also detected in Sertoli cells and interstitial Leydig cells in control tissues. Although cytoplasmic staining was faint when compared with spermatogenic cells, it still seemed more intense than in Sertoli cells from the SCOS patient’s tissue. To confirm these results, staining with two other experimental FANCM antibodies (Supplementary Figure S3) was performed with similar results, although the staining intensity was slightly weaker than with commercial antibodies.

### 3.4. Localization of FANCM Protein in the Astrocytoma

**Figure 3:**
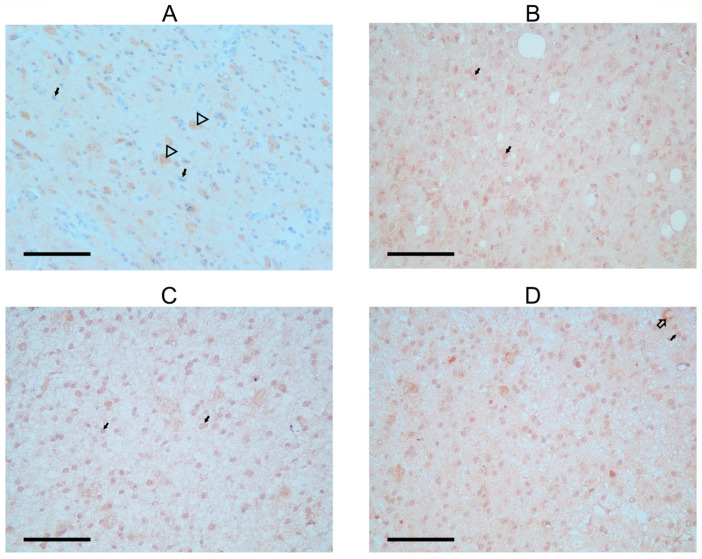
Immunohistochemical detection of FANCM protein in astrocytoma biopsies. (**A**) FANCM staining in astrocytoma of a SCOS patient; arrowhead shows positivity is detected in neurons, and black arrows show faint to no staining in tumor cells. (**B**) Second biopsy of astrocytoma from SCOS patient after radiation treatment; black arrows show FANCM staining, a signal of stronger intensity in glial and tumor cells. (**C**,**D**) Two control astrocytoma patients with the same tumor characteristics as the SCOS patient. Black arrows show FANCM staining with more positivity in the area around the tumor cells. Arrows with black outlines show cytoplasmic staining is also present in neurons and around blood vessels (**C**).

In the patient’s astrocytoma, astrocytic tumor cells displayed a faint, primarily negative FANCM staining signal, with just a few cells stained around the nucleus. On the other hand, a stronger FANCM signal was found in the cytoplasm of neurons that remained within the tumor tissue (Figure 3A; Table 1). Staining was also observed in the neuropil, a space between neuronal and glial cell bodies composed of dendrites, axons, glial cell processes, and microvasculature. In all cases, neuropil showed some staining that could be the background. Astrocytomas from the control group (Figure 3C,D) had stronger signal intensity in tumor cells than in the SCOS patient, while the FANCM signal in the neurons shared a similar expression pattern.

In the second biopsy of the SCOS patient, FANCM expression was detected again in the cytoplasm of tumor cells (Figure 3B). The staining intensity was faint but stronger when compared with the staining observed before radiation. Two additional experimental antibodies confirmed all observed patterns (Supplementary Figure S5).

## 4. Discussion

In this study, we examined the genetics of a patient diagnosed with non-obstructive azoospermia with the SCOS phenotype who was later diagnosed with a central nervous system tumor: a diffuse astrocytoma.

The exome sequencing led us to find two heterozygous missense variants (Leu57Phe and Gln317Arg) in the FANCM gene in a compound form. Previously, FANCM loss-of-function variants have been linked to severe spermatogenesis failure in azoospermia patients [26,27]. Furthermore, although it appears that FANCM does not cause FA, a cancer-predisposition syndrome, but rather, FA-like syndrome [35,46], pieces of evidence are emerging that correlate its dysfunctionality to cancer predisposition. Homozygous FANCM loss-of-function variants have already been observed in patients with early-onset cancers who also seem to be highly sensitive to chemotherapies [35,47]. Moreover, some monoallelic FANCM variants are clearly associated with a predisposition to breast cancer [32,33,34] and possibly with some other cancers [36,37].

Hence, we find it important to raise awareness regarding how compound heterozygous FANCM variants in the SCOS patient affected his complex clinical picture, which combined spermatogenesis failure (SCOS) and a cancer diagnosis.

The reported FANCM variants in male infertility studies are either biallelic recessive loss-of-function or frameshift variants [26,27]. Herein, we report the same gonadal impairment with compound heterozygous missense variants. Both variants have been reported in ClinVar, although with uncertain significance or conflicting interpretations of their pathogenicity. Both variants had high Combined Annotation-Dependent Depletion (CADD) scores, implying their functional effect.

Both variants were found at the N-terminus, next to or within the ATPase/helicase domain of FANCM, which has translocase activity (residues 64–684). Structurally, the ATPase/helicase domain is composed of two RecA-like folds separated by an insertion domain (residues 298–433) [48] characteristic of SF2 helicases [49,50,51]. Mutations in the insertion domain were shown to affect the protein translocase function in in vitro systems [52,53]. Thus, it is possible that Gln317Arg mutation inside the insertion domain impaired the FANCM protein’s function in maintaining genomic stability during cell division, e.g., promoting the recovery of stalled replication forks [23,54]. The other variant (Leu57Phe) is located near the start of the helicase domain in a still non-resolved structure of the N-terminus of the protein, which limited the interpretation possibilities. However, this variant was previously reported in a breast cancer patient as a monoallelic VUS [33].

Related to the role of FANCM N-terminus mutations in SCOS development and cancer, it was shown that a mouse with a single amino acid change in the helicase domain also exhibited progressive germ cell depletion to a near SCOS phenotype, similar in FANCM knockout mice [55,56]. The authors suggested a causative defect in spermatogonial proliferation or even mitotic renewal. FANCM mutations could compromise the ability to repair DNA damage during cell division, potentially leading to apoptosis and consequential spermatogenesis failure. Recently, in knockout models, it was discovered that FANCM plays a significant role in limiting the number of meiotic crossovers during gamete formation and maintaining germ cell integrity, with an essential role in activating cell cycle checkpoints and apoptotic pathways. Any disruption of these primary mechanisms can lead to genomic instability, subsequently resulting in chromosomal abnormalities and germ cell loss [57].

Moreover, FANCM’s recruitment into ultrafine bridges, which link sister chromatids at DNA fragile sites in the telophase, has also been demonstrated [33,58]. Failure in telophase resolution thus might explain the SCOS phenotype observed here, as well as cancer susceptibility due to a possible ploidy defect in the meiotic cell cycle. Finally, the possibility that two missense variants in the N-terminus could lead to cancer is supported by the same mouse studies where a single mutation introduced in the helicase domain of FANCM also led to a cancer-prone phenotype (elevated sister-chromatin exchange, tumor susceptibility, and premature senescence) [56]

Although the nuclear role of FANCM has been extensively studied, our study showed that FANCM was mainly present in the cytoplasm of both the testes and astrocytoma of the patient, as well as in the control’s prostate cells. This was confirmed by three different antibodies, as well as by other studies on human samples. A comprehensive study of the FANC family member’s expression in human testes showed FANCM localization in the cytoplasm of Sertoli cells and the cytoplasm and Golgi of spermatogonia through to early pachytene spermatocytes [59]. This differs slightly from our study, as we noticed a stronger expression in spermatogonia compared with spermatocytes, though we could not notice Golgi localization. Despite this, one explanation for this cytoplasmic and/or Golgi localization comes from a study demonstrating that, in mammalian mitotic cells, DNA damage causes the Golgi to fragment and disperse throughout the cytoplasm, leading to the amplification of the DNA damage response [60]. Hence, the presence of FANCM and some other FANC proteins found in Golgi (such as FANCD2) raises the possibility that the newly synthesized FANC proteins may undergo post-translational modification and are stored before being distributed to sites of DNA damage foci at appropriate time points of germ cell development [59].

Interestingly, we found FANCM protein for the first time in the cytoplasm of non-dividing neuronal cells. This could be explained by the necessity of post-mitotic cells, such as neurons, in repairing interstrand crosslinks (ICLs) independently of replication. ICLs are cytotoxic DNA lesions that are induced in DNA from exogenous sources but may also arise endogenously, and their repair is critical for cellular homeostasis [61]. Another reason for FANCM’s presence in neurons could come from recent evidence of FANC family activity in the clearance of damaged mitochondria, which is a role very independent of its function in nuclear DNA damage repair [62]. Mitophagy is critical to maintaining neuronal homeostasis [63], which might explain the cytoplasmic expression of FANCM in neuronal cells.

Regarding staining intensity, FANCM showed not only very low staining in the testicular Sertoli cells of the SCOS patient but also in the matching astrocytoma, where FANCM showed very low expression in neoplastic astrocytes and with visibly lower intensity in comparison with control astrocytomas with the same pathological characteristics. We speculate that changed amino acid structures in important domains caused a decrease in FANCM expression and function but not complete absence. However, this malfunctioning of FANCM could increase genome instability during glial cell replication and lead to the final development of astrocytomas. A major limitation of our study is the lack of a functional study to determine the real impact of the detected variants on SCOS and the possibility that other somatic mutations could affect astrocytoma development.

FANCM mutation carriers do not develop classical Fanconi anemia; they develop Fanconi-like anemia, often without clinical symptoms [28]. However, it was shown here that mutations in FA genes, as well as in FANCM, are related to the hypersensitivity of these patients to DNA-damaging agents such as chemotherapy and radiotherapy, which might lead to poor treatment outcomes. Thus, we find this case suggests that genetic counseling should be applied to azoospermia patients diagnosed with FANC mutations, with proposals for the multidisciplinary monitoring of these men in terms of regular screenings for cancer or hematologic abnormalities and other potential complications. In the sequencing era, these actions would be not only essential for early cancer detection and intervention but also for delivering strategic, personalized treatments, such as dose reductions or alternative regimens of chemotherapy and/or radiotherapy. Awareness of FANCM mutations can thus result in life-saving measures for patients and their family members.

## Figures and Tables

**Table 1:** Intensity and localization of FANCM immunohistochemical signal in control testicular tissue and testicular/astrocytoma tissue of SCOS patient.

Tissue	Cell Type	Localization	Intensity
Control testis	Spermatogonia	Cytoplasm	+++
	Primary spermatocytes	Cytoplasm	++
	Secondary spermatocytes	Cytoplasm	++
	Spermatids	Cytoplasm	+++
	Sertoli cells	Cytoplasm	+
	Leydig cells	Cytoplasm	+
Patient’s testis	Sertoli cells	Cytoplasm	−/+
	Leydig cells	Cytoplasm	+
Control astrocytoma tissue	Neurons	Cytoplasm/nucleus	++
	Tumor cells	Cytoplasm	+
Patient’s astrocytoma tissue	Neurons	Cytoplasm/nucleus	++
	Tumor cells	Cytoplasm	−/+

No signal −, weak intensity +, moderate intensity ++, and strong intensity +++.

## Data Availability

Data supporting this study cannot be made available based on information from the patient’s informed consent form. Written informed consent was obtained from the patient(s) for their anonymized information to be published in this article.

## Supplementary Materials

The following supporting information can be downloaded at https://www.mdpi.com/article/10.3390/genes15060707/s1: Figure S1: Testicular histology of FANCM patient’s Sertoli cells only. Figure S2: Histology of FANCM patient’s astrocytoma. Figure S3: Immunohistochemical detection of FANCM with two additional antibodies in tubules of FANCM patient and control with complete details. Figure S4: Immunohistochemical detection of FANCM with two additional antibodies in astrocytoma of FANCM patient and control astrocytoma. Figure S5: Sanger sequencing of patient’s mother’s FANCM. Table S1: Top variants in father and son with combined Exomiser scores greater than 0.9.
