# Supplementary figures and images for: FANCM Gene Variants in a Male Diagnosed with Sertoli Cell-Only Syndrome and Diffuse Astrocytoma
Source: Genes (Basel). 2024 May 28;15(6):707. doi: 10.3390/genes15060707 (PMC11202954; doi:10.3390/genes15060707)

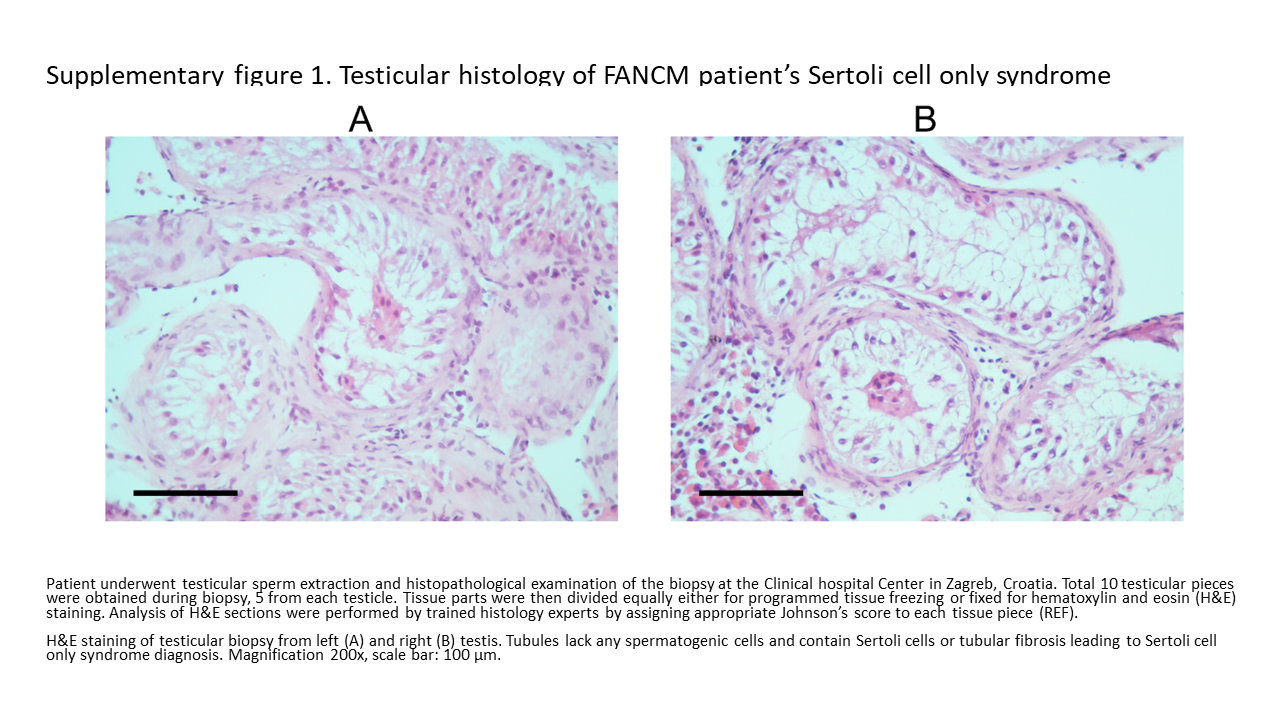

Supplement: Supplementary file 1 [file genes-15-00707-s001.zip › Supplementary Materials/Figure S1 Testicular histology of FANCM patient’s Sertoli cell only.PNG]

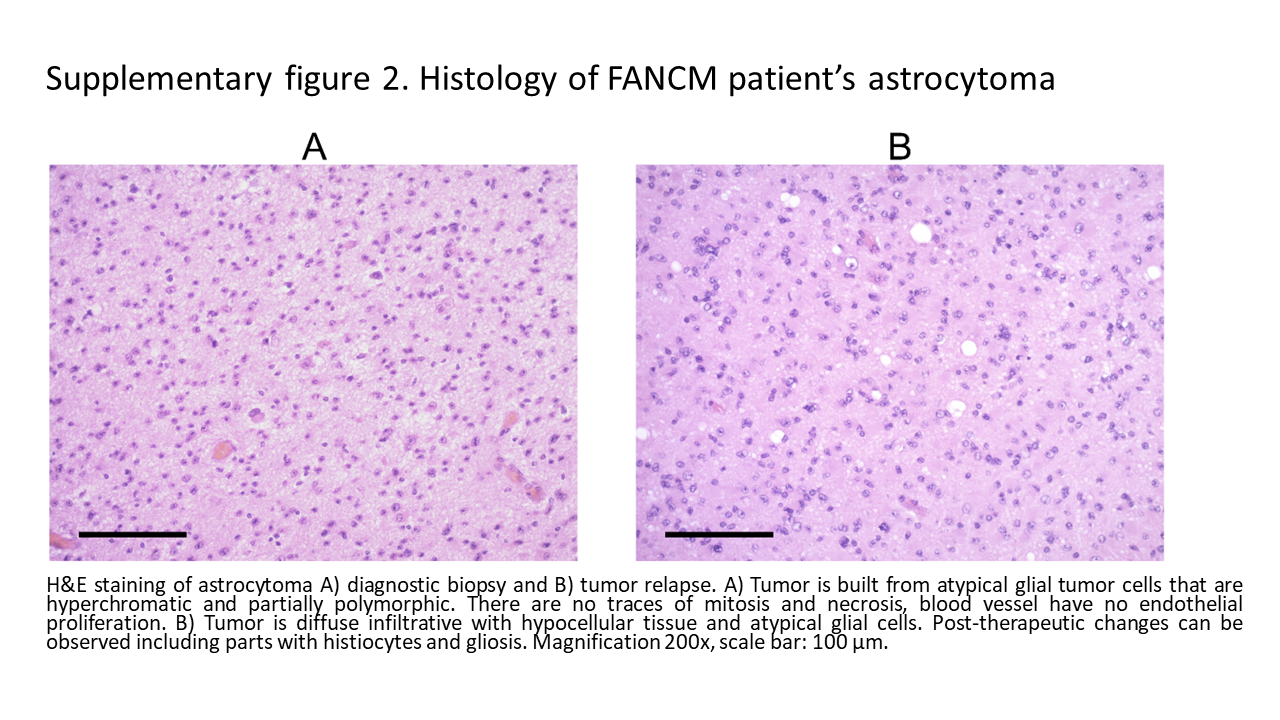

Supplement: Supplementary file 1 [file genes-15-00707-s001.zip › Supplementary Materials/Figure S2 Histology of FANCM patient’s astrocytoma.PNG]

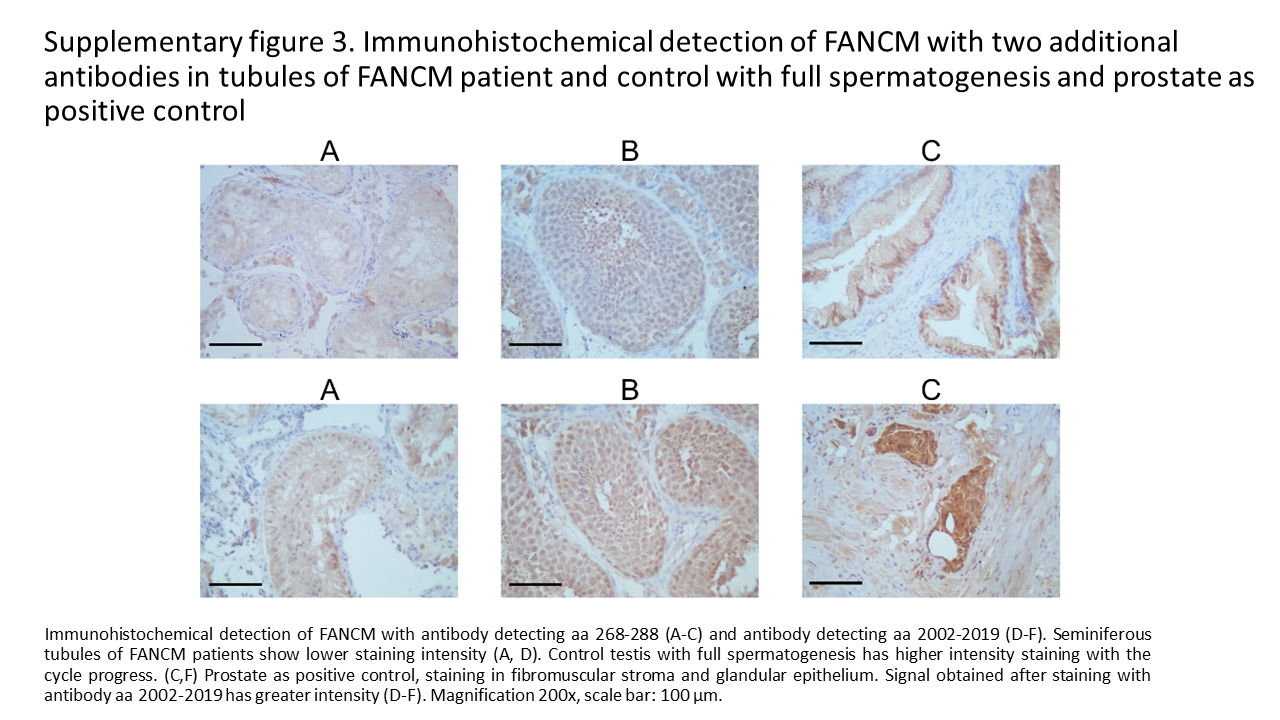

Supplement: Supplementary file 1 [file genes-15-00707-s001.zip › Supplementary Materials/Figure S3 Immunohistochemical detection of FANCM with two additional antibodies in tubules of FANCM patient and control with full.PNG]

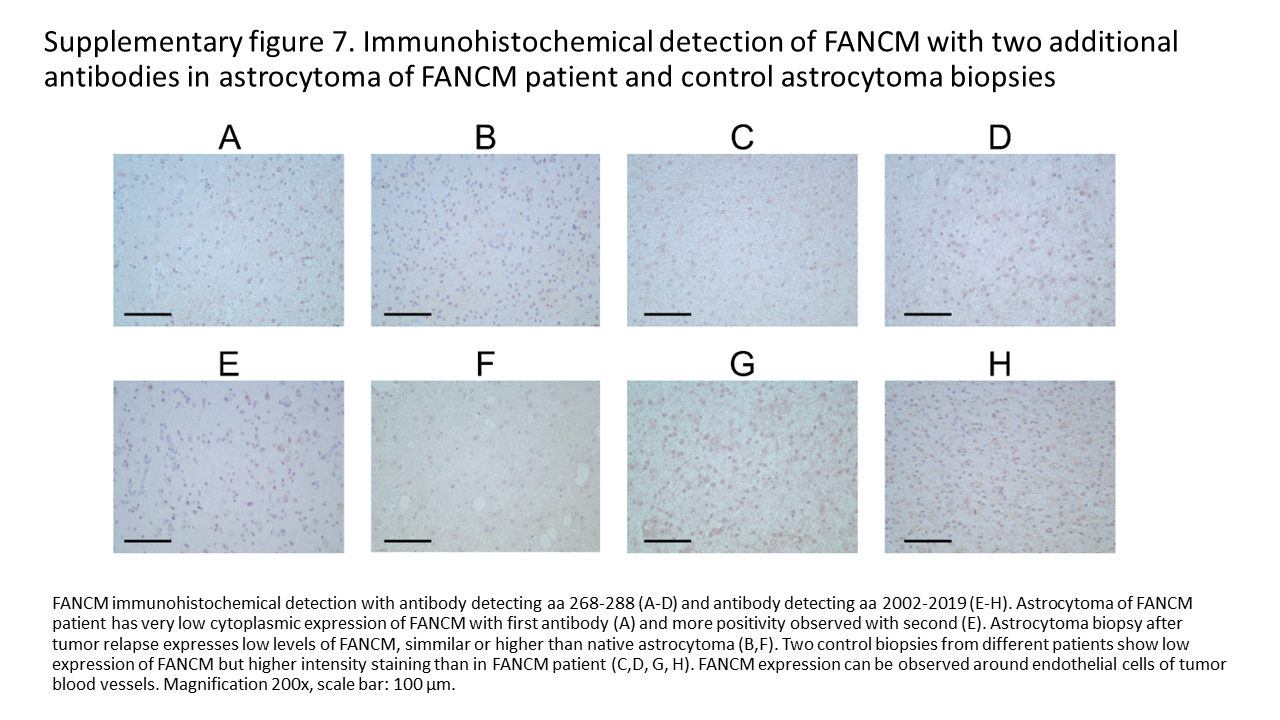

Supplement: Supplementary file 1 [file genes-15-00707-s001.zip › Supplementary Materials/Figure S4 Immunohistochemical detection of FANCM with two additional antibodies in astrocy-toma of FANCM patient and control astrocytoma.PNG]

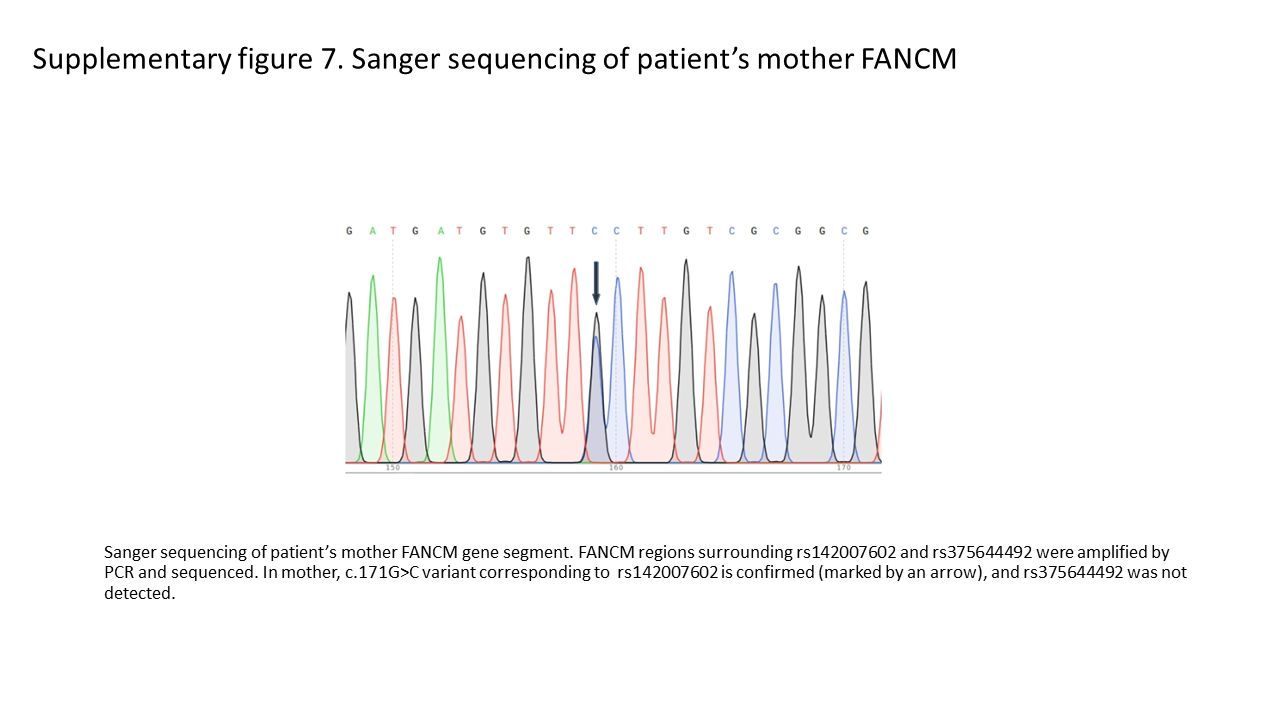

Supplement: Supplementary file 1 [file genes-15-00707-s001.zip › Supplementary Materials/Figure S5 Sanger sequencing of patient’s mother FANCM.PNG]
